# Integrated genomic and metabolomic analysis reveals the biocontrol potential of endophytic *Bacillus velezensis* NS13 against *Fusarium* species in *Lonicera macranthoides*

**DOI:** 10.1186/s12866-025-04551-x

**Published:** 2026-01-05

**Authors:** Junpeng Qi, Zhong Chen, Sheng’e Lu, Li Liu, Han Wang, Wei Zhuo, Yuqi Wang, Meisen Yang, Hongxu Zhou, Yin Yang, Fengming Ren

**Affiliations:** 1 Chongqing Institute of Medicinal Plant Cultivation, Nanchuan, Chongqing, 408435 China; 2 School of Chinese Materia Medica, Chongqing College of Traditional Chinese Medicine, Bishan , Chongqing, 402760 China; 3 Traditional Chinese Medicine Industry Center of Xiushan County, Xiushan, Chongqing, 409900 China; 4 CQMPA Key Laboratory for Quality Control and Evaluation of Traditional Chinese Medicine, Chongqing Institute for Food and Drug Control, Yubei, Chongqing, 401120 China; 5 Chongqing Traditional Chinese Medicine Hospital, Yuzhong, Chongqing, 400011 China; 6 Planting Industry Development Service Center of Zunyi City, Zunyi, Guizhou 563000 China

**Keywords:** *Fusarium* species, *Bacillus velezensis* NS13, Genomics, *Lonicera macranthoides* root rot, Metabolomics

## Abstract

*Fusarium* species are major fungal pathogens causing root rot in *Lonicera macranthoides* and other agriculturally and medicinally important plants. They exhibit a broad host range and high pathogenicity, leading to yield losses, reduced quality, and plant mortality. Current control measures rely primarily on chemical pesticides, with few sustainable biological options available. This study compared rhizosphere microbial diversity between healthy and diseased *Lonicera macranthoides*, revealing increased pathogenic fungi abundance (*Fusarium*, *Plectosphaerella*, *p* < 0.01) and reduced beneficial fungi abundance (*Trichoderma*) along with significantly lower Chao1 and Shannon diversity indices (*p* < 0.05). An endophytic *Bacillus velezensis* strain, NS13, was isolated from healthy roots. Plate confrontation assays showed strong inhibition of *Fusarium oxysporum* from *L. macranthoides* and other *Fusarium* species (*Fusarium solani*, *Fusarium graminearum*, *Fusarium fujikuroi*). The 3.95 Mb genome encoded 4,060 proteins, including 96 biocontrol-related genes. AntiSMASH identified 15 biosynthetic gene clusters, including antifungal (fengycin, surfactin), antibacterial (bacillaene, difficidin), and other bioactive metabolites (bacilysin, bacillibactin), alongside seven potentially novel clusters. The presence of these BGCs was further corroborated by LC-MS/MS metabolomic profiling, which detected multiple corresponding antifungal metabolites, including cyclic dipeptides, fatty acid amides (e.g., erucamide), and oleanolic acid. These results demonstrate soil microbial dysbiosis in *L. macranthoides* affected by root rot and confirm the broad-spectrum anti-*Fusarium* potential of NS13, highlighting its promise as a biocontrol resource against *Fusarium* pathogens in medicinal plants. The findings provide both theoretical insights and practical guidance for developing sustainable biocontrol strategies against *Fusarium* and other soil-borne pathogens, benefiting both the scientific community and agricultural practitioners.

**Supplementary Information:**

The online version contains supplementary material available at 10.1186/s12866-025-04551-x.

## Background

*Fusarium* species are ubiquitous and highly destructive fungal pathogens with broad host specificity, systemic infection capabilities, and strong environmental adaptability [1]. They rapidly infect the roots, stem bases, and vascular systems of various crops, causing devastating diseases such as root rot, stem rot, and wilting [2]. The thick-walled chlamydospores of *Fusarium* can persist in soil for extended periods and produce mycotoxins, such as fusaric acid, posing severe threats to agricultural safety and human health [3, 4]. Their robust sporulation and dissemination capacities enable regional or even trans-regional spread through infected plants, irrigation water, wind, agricultural practices, or seedling trade, leading to rapid disease outbreaks [5]. The covert and systemic nature of *Fusarium* infections makes early detection challenging and eradication difficult, often resulting in continuous cropping obstacles [6]. The high virulence, pathogenicity, and transmissibility of *Fusarium* species render them among the most challenging soil-borne fungal pathogens to control in agricultural ecosystems [7]. 

*Lonicera macranthoides*, an important medicinal plant in China [8], faces increasing challenges from root rot during cultivation, which severely impacts yield and quality, posing a critical bottleneck to the industry’s sustainable development. *Lonicera* species, closely related to *L. macranthoides* [9, 10], are similarly affected by root rot during cultivation. Research indicates that the genus *Fusarium* induces a range of symptoms in host plants, including wilting, root rot, and bulb rot, resulting in diseases with significant economic impacts [11]. Additionally, *Fusarium* species broadly affect other plants [12], such as *Polygonatum odoratum* and *Polygonatum cyrtonema*, in which *Fusarium oxysporum* causes substantial tuber yield losses [13, 14]. Root rot in medicinal crops like *Angelica sinensis* [15], *Panax ginseng* [16], *Panax notoginseng* [17], and *Codonopsis pilosula* [18] is often linked to *Fusarium oxysporum* and *Fusarium solani*, with disease mechanisms closely tied to mycotoxin production, such as fusaric acid, underscoring the widespread threat of *Fusarium* to medicinal plants [19].

Currently, *Fusarium* disease control primarily relies on chemical fungicides, but their prolonged use leads to environmental pollution, pesticide residues, pathogen resistance, and soil microbial dysbiosis, necessitating green, safe, and effective alternatives [20]. Endophytic bacteria, which naturally colonize host tissues and form stable symbiotic relationships within their environment, offer unique advantages in biocontrol [21]. Their mechanisms include: 1) resource competition, whereby siderophores and alkaline phosphatases compete with pathogens for carbon, nitrogen, and iron [22]; 2) direct antibiosis through non-ribosomal peptides, polyketides, and chitinases [23]; 3) induced resistance via activation of phytoalexin synthesis and cell wall reinforcement; [24] and 4) growth promotion through nitrogen fixation and phosphate solubilization, enhancing plant nutrition and disease resistance [25]. These synergistic mechanisms provide a robust foundation for the application of endophytic bacteria in disease control.

*Bacillus velezensis*, a member of the *Bacillus amyloliquefaciens* subgroup, is recognized for its broad-spectrum antimicrobial properties and stable biocontrol efficacy [26]. Its genome is rich in non-ribosomal peptide synthetase (NRPS) and polyketide synthase (PKS) gene clusters [27], enabling the synthesis of antifungal and antibacterial metabolites such as iturin, fengycin, bacillomycin, difficidin, and macrolactin [28]. Studies show that *B. velezensis* FZB42 and its derivatives effectively suppress *Fusarium*-related diseases, including wheat head blight [29], tomato wilt [30], and pepper root rot [31], while stably colonizing the rhizosphere or root tissues for sustained biocontrol [29]. This species also regulates plant hormone balance, enhances stress tolerance, and improves nutrient uptake, making it an ideal biocontrol and growth-promoting agent [32]. However, its application in medicinal plants, particularly for *L. macranthoides* root rot, remains underexplored.

Addressing this research gap, this study isolated *B. velezensis* NS13 from *L. macranthoides* roots and systematically evaluated its inhibitory effects against *Fusarium oxysporum*, *Fusarium fujikuroi*, *Fusarium solani*, and *Fusarium graminearum*, the causal agents of root rot. The antifungal mechanisms were elucidated at the genetic and metabolic levels, providing a valuable microbial resource and theoretical basis for the green control of *L. macranthoides* root rot and providing new insights for sustainable disease management in medicinal plants.

## Methods

### Plant and soil materials

Healthy and root rot-affected *L. macranthoides* plants and their rhizosphere soil were collected from a cultivation base in Yun’ai Village, Zhongling Town, Xiushan County, Chongqing, China (28°47′N, 108°59′E). Diseased plants exhibited typical root rot symptoms (root decay, plant wilting). Soil samples were collected using a five-point sampling method within a 10–50 cm radius from the plant’s main stem base, removing surface litter and stones, and excavating to a depth of ~ 20 cm to collect 0–2 mm soil tightly adhering to roots. Samples from each treatment (healthy/diseased) were thoroughly mixed, stored in sterile bags, kept in an icebox, and rapidly transported to the laboratory. Each treatment included three biological replicates.

### Soil microbial DNA extraction and high-throughput sequencing

Total soil DNA was extracted using a DNA extraction kit (Tiangen Biotech, Beijing, China) following the manufacturer’s instructions. Fungal community analysis targeted the ITS1 region using primers ITS1F (5’-CTTGGTCATTTAGAGGAAGTAA-3’) and ITS2R (5’-GCTGCGTTCTTCATCGATGC-3’) for PCR amplification. The 25 µL reaction system included 12.5 µL 2× Taq PCR MasterMix (Vazyme, China), 1 µL of each primer (10 µmol/L), 2 µL DNA template (50 ng/µL), and 8.5 µL nuclease-free water. The PCR program was: 95 °C for 5 min; 35 cycles of 95 °C for 30 s, 55 °C for 30 s, 72 °C for 30 s; and 72 °C for 10 min. PCR products were verified by 2% agarose gel electrophoresis, purified using a Qiagen Gel Extraction Kit (Qiagen, Germany), and sequenced on the Illumina MiSeq platform (2 × 250 bp) by Shanghai Sangon Biotech Co., Ltd. A 5–10% PhiX control was added to ensure sequencing quality.

### Bioinformatics and statistical analysis

Raw sequencing data were quality-controlled using QIIME2, removing low-quality sequences (quality score < 20, length < 200 bp) and chimeras to obtain clean tags. Operational taxonomic units (OTUs) were clustered at 97% similarity using VSEARCH [33], with taxonomic annotation performed against the UNITE database (v8.3) [14]. Alpha diversity indices (Chao1 richness, Shannon diversity) were calculated using VSEARCH. Differences in alpha diversity between healthy and diseased rhizosphere soils were assessed using t-tests (*p* < 0.05). The relative abundance of the top 10 dominant genera was visualized using bar plots generated with the R package ggplot2(https://ggplot2.tidyverse.org/).

### Endophytic bacteria isolation

Healthy *L. macranthoides* roots were collected from the Yun’ai Village cultivation base. Surface soil was removed using a sterile scalpel, followed by surface sterilization: 75% ethanol for 2 min, 3% sodium hypochlorite for 5 min, and three rinses with sterile water. Sterilized root segments were cut into 0.5 cm pieces, ground in a sterile mortar, and suspended in 10 mL 0.85% sterile saline. The suspension was serially diluted (10⁻³–10⁻⁵), and 100 µL of each dilution was spread onto LB agar plates (tryptone 10 g/L, yeast extract 5 g/L, NaCl 10 g/L, agar 15 g/L, pH 7.0) and incubated at 28 °C for 48 h. Single colonies were selected based on morphology, purified by streaking three times, and stored at 4 °C.

### Physiological, biochemical, and molecular identification of strain NS13

Strain NS13 was cultured on LB agar at 30 °C for 24 h, and colony morphology (color, shape, margin) was observed. Gram staining was performed using a kit (Solarbio, China) and observed under a microscope. For scanning electron microscopy (SEM), NS13 was cultured in LB liquid medium (37 °C, 150 rpm) for 48 h, centrifuged (4,500 g, 5 min, 4∘C), then washed with PBS, resuspended in 2.5% glutaraldehyde fixative, fixed at room temperature for 2 h, and stored at 4 °C.

For molecular identification, genomic DNA was extracted using a bacterial DNA extraction kit (Omega Bio-tek, USA) and quantified with a TBS-380 fluorometer (Turner BioSystems, USA). The 16S rRNA gene sequences were compared against the GenBank database using BLAST to confirm taxonomic status.

Representative isolates exhibiting *Bacillus*-like characteristics were subjected to molecular identification. The 16S rRNA gene sequence was analyzed using BLAST (Basic Local Alignment Search Tool) against the NCBI (National Center for Biotechnology Information) database to determine the phylogenetic affiliation of the isolate. Based on its typical *Bacillus*-like morphological traits, the strain was inferred to possess a biocontrol background and potential antifungal activity. Consequently, strain NS13 was selected as a candidate for subsequent studies on the biological control of *Fusarium* species.

### Plate confrontation assay of NS13 against *Fusarium* species

Four representative plant-pathogenic *Fusarium* strains were used in this study. Three reference strains were obtained from the BeNa Culture Collection (BNCC) and one strain was isolated previously in our laboratory from the rhizosphere soil of *Lonicera macranthoides* exhibiting root-rot symptoms (details provided in Table S1). All strains were routinely cultured on potato dextrose agar (PDA; potato 200 g L⁻¹, glucose 20 g L⁻¹, agar 15 g L⁻¹; pH 6.0) and grew stably under these conditions. Fungal strains were maintained on LB slants at 4 °C for short-term storage and preserved in 20% (v/v) glycerol at − 80 °C for long-term storage.

The antagonistic ability of NS13 was evaluated using a plate confrontation assay. NS13 was cultured in LB liquid medium (28 °C, 150 rpm) to an OD₆₀₀ of 1.0. *Fusarium* strains were grown on PDA plates until the colony diameter reached ~ 8 cm, and a 6 mm diameter fungal disc was taken from the colony edge and placed at the center of a new PDA plate. Four 1.0 µL drops of NS13 culture were symmetrically inoculated 2.0 cm from the fungal disc. The control group (CK) received equal volumes of LB medium. Each treatment had three replicates. Plates were incubated at 28 °C until the control colony diameter reached 8 cm. Colony diameters (average of the longest and shortest perpendicular axes, in mm) were measured for both treatment and control groups [34]. Inhibition rate was calculated as: Inhibition rate (%) = [(Dc – Dt)/Dc] × 100, where Dc is the control colony diameter and Dt is the treatment colony diameter. Data were processed in Microsoft Excel, and significant differences were analyzed using Student’s t-test in SPSS (v26.0, IBM Corp., USA) (*p* < 0.05).

### Whole-genome sequencing and assembly of NS13

*B. velezensis* NS13 was cultured in LB liquid medium (37°C, 150 rpm). Genomic DNA (gDNA) was extracted using the Invitrogen PureLink^®^ Genomic DNA Extraction Kit (Thermo Fisher Scientific, USA), with concentration and purity assessed using a NanoDrop ND-1000 spectrophotometer, followed by purification with the Zymo Quick-DNA Kit (Zymo Research, USA). For next-generation sequencing (NGS), a ~ 400 bp insert paired-end library was constructed: gDNA was fragmented using a Covaris sonicator, end-repaired with T4 DNA polymerase, A-tailed at the 3’ end, ligated with adapters, size-selected by gel electrophoresis, and amplified with indexing PCR. The library was quality-checked using an Agilent Bioanalyzer 2100 and sequenced on the Illumina platform (150 bp paired-end) by Shanghai Biozeron Biotech Co., Ltd. For PacBio sequencing, an SMRTbell library was prepared using the Express Template Prep Kit 2.0, with fragments > 8 kbp selected using BluePippin, and sequenced on the Sequel II platform.

Genome assembly was performed as follows: NGS data were quality-controlled using Trimmomatic (v0.39) [35] with parameters SLIDINGWINDOW:4:15 MINLEN:75, PacBio long reads were error-corrected, and assembly was conducted using Unicycler (v0.5.0) [36] with default parameters, followed by genome circularization using Circlator (v1.5.5) [37].

### Phylogenomic analysis of NS13

The reference genome of *B. velezensis* FZB42 was downloaded from NCBI (https://www.ncbi.nlm.nih.gov). Phylogenomic analysis was performed using the Type Genome Server (TYGS, http://tygs.dsmz.de), constructing a maximum likelihood phylogenetic tree based on genome BLAST distance with 1,000 bootstrap replicates, visualized using PhyD3. Digital DNA-DNA hybridization (dDDH) was calculated using the Genome-to-Genome Distance Calculator 3.0 (GGDC, https://ggdc.dsmz.de/ggdc.php) with formula 2 (100 replicates), with a species threshold of ≥ 70%. Average nucleotide identity (ANI) was computed using JspeciesWS (https://jspecies.ribohost.com/jspeciesws/) with the BLAST algorithm (ANIb), with a species threshold of ≥ 95%.

### Annotation of antibacterial genes and secondary metabolite biosynthetic gene clusters

NS13 protein-coding genes were annotated using public databases (NR, Swiss-Prot, COG, GO, KEGG). Biocontrol-related genes were categorized into four functional groups: (1) Resource Competition Genes (RCG), mediating competition for space and nutrients (e.g., siderophore synthesis/transport, alkaline phosphatase-related genes); (2) Antibacterial Activity Genes (AAG), encoding antibiotics (Nonribosomal Peptide Synthetases/Polyketide Synthases, NRPS/PKS), antimicrobial peptides, and cell wall-degrading enzymes (e.g., chitinase, lysozyme-related genes); (3) Induced Resistance Genes (IRG), activating host defense mechanisms (e.g., phytoalexin synthesis, cell wall reinforcement-related genes); and (4) Stress-Resistance Genes (SRG), enhancing plant resilience through nitrogen fixation (e.g., nifH/nifD) or phosphate solubilization(e.g., gcd, pqqABC/phoA/phoD genes). Secondary metabolite biosynthetic gene clusters (BGCs) were predicted using antiSMASH (v4.1.0) [38] and compared with *B. velezensis* FZB42.

### LC-MS/MS analysis of NS13 metabolites

NS13 fermentation broth was centrifuged (10,000 × g, 10 min, 4 °C) to remove solids, and the supernatant was collected. The pH was adjusted to 2.0 with HCl to optimize metabolite extraction or precipitation. Samples were incubated at 4 °C overnight to promote precipitation, re-centrifuged (10,000 × g, 10 min, 4 °C), and the precipitate was extracted with acetonitrile. The extract was concentrated under reduced pressure, lyophilized, reconstituted in methanol: water (80:20, v/v), and filtered through a 0.22 μm membrane to remove particles.

LC-MS/MS analysis was performed using a reverse-phase C18 column (ACQUITY UPLC BEH C18, 2.1 × 100 mm, 1.7 μm). The mobile phase consisted of water + 0.1% formic acid (A) and acetonitrile + 0.1% formic acid (B), with a gradient of 0–2 min, 5% B; 2–20 min, 5%–95% B; 20–25 min, 95% B; 25–27 min, return to 5% B. The flow rate was 0.3 mL/min, column temperature was 40 °C, and injection volume was 3 µL. Mass spectrometry was conducted on a high-resolution Thermo Q Exactive HF-X in positive ion mode, with a full scan range of m/z 150–2000 (resolution 70,000), using data-dependent acquisition (DDA) or targeted parallel reaction monitoring (PRM), HCD fragmentation (NCE 25–35), MS/MS resolution of 17,500, and dynamic exclusion of 15 s. Raw data were processed using Xcalibur and Compound Discoverer 3.3, with metabolite identification performed against GNPS, MassBank, and PubChem databases.

## Results

### Soil fungal community diversity analysis in root rot-affected *L. macranthoides*

**Fig. 1:**
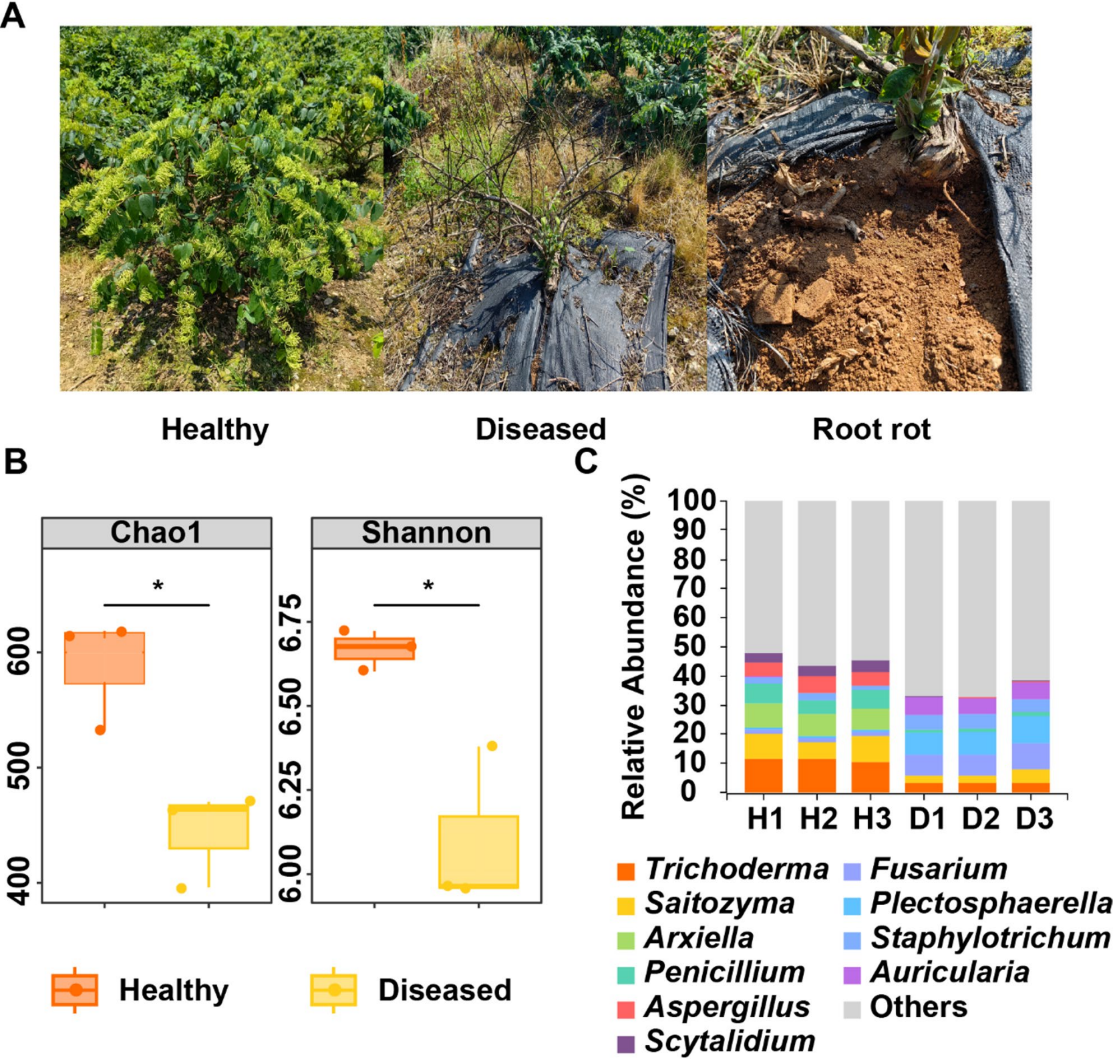
Morphological and microbial community characteristics of *L. macranthoides* root rot. **A** Phenotype of root rot. **B** Alpha diversity indices (Chao1 and Shannon) of rhizosphere microbial communities in healthy and diseased plants. **C** Genus-level composition of rhizosphere microbial communities in healthy and diseased plants

Phenotypic observations of *L. macranthoides* root rot (Fig. 1A) showed yellow-brown decay of fine roots progressing to main roots, with later stages exhibiting black-brown softening, leaving only the xylem, accompanied by leaf yellowing, stem base browning, growth cessation, and eventual plant death. Alpha diversity analysis (Fig. 1B) revealed significantly lower Chao1 (richness) and Shannon (diversity) indices in the rhizosphere fungal communities of diseased plants compared to healthy ones (*p* < 0.05), indicating a simplified microbial community structure and disrupted homeostasis. This suggests that root rot significantly alters rhizosphere microbial community structure and diversity.

Genus-level composition analysis (Fig. 1C) revealed a marked shift in community structure. Healthy plants’ rhizospheres were dominated by beneficial genera such as *Trichoderma* (biocontrol function [39]), *Saitozyma*, *Arxiella*, *Penicillium*, and *Aspergillus* (top 5), diseased plants’ rhizospheres showed a significant increase in pathogenic genera, including *Fusarium*, *Plectosphaerella*, *Auricularia*, and *Staphylotrichum*. Quantitative comparisons (Figure S1A) showed significantly reduced abundances of *Trichoderma*, *Saitozyma*, *Arxiella*, *Penicillium*, *Aspergillus*, and *Scytalidium* in diseased plants, while *Fusarium*, *Plectosphaerella*, *Auricularia*, and *Staphylotrichum* (Figure S1B) were significantly enriched (*p* < 0.01), indicating a strong association between root rot and the enrichment of pathogenic fungi and depletion of beneficial microbes.

### Isolation and identification of *Bacillus velezensis* NS13

**Fig. 2:**
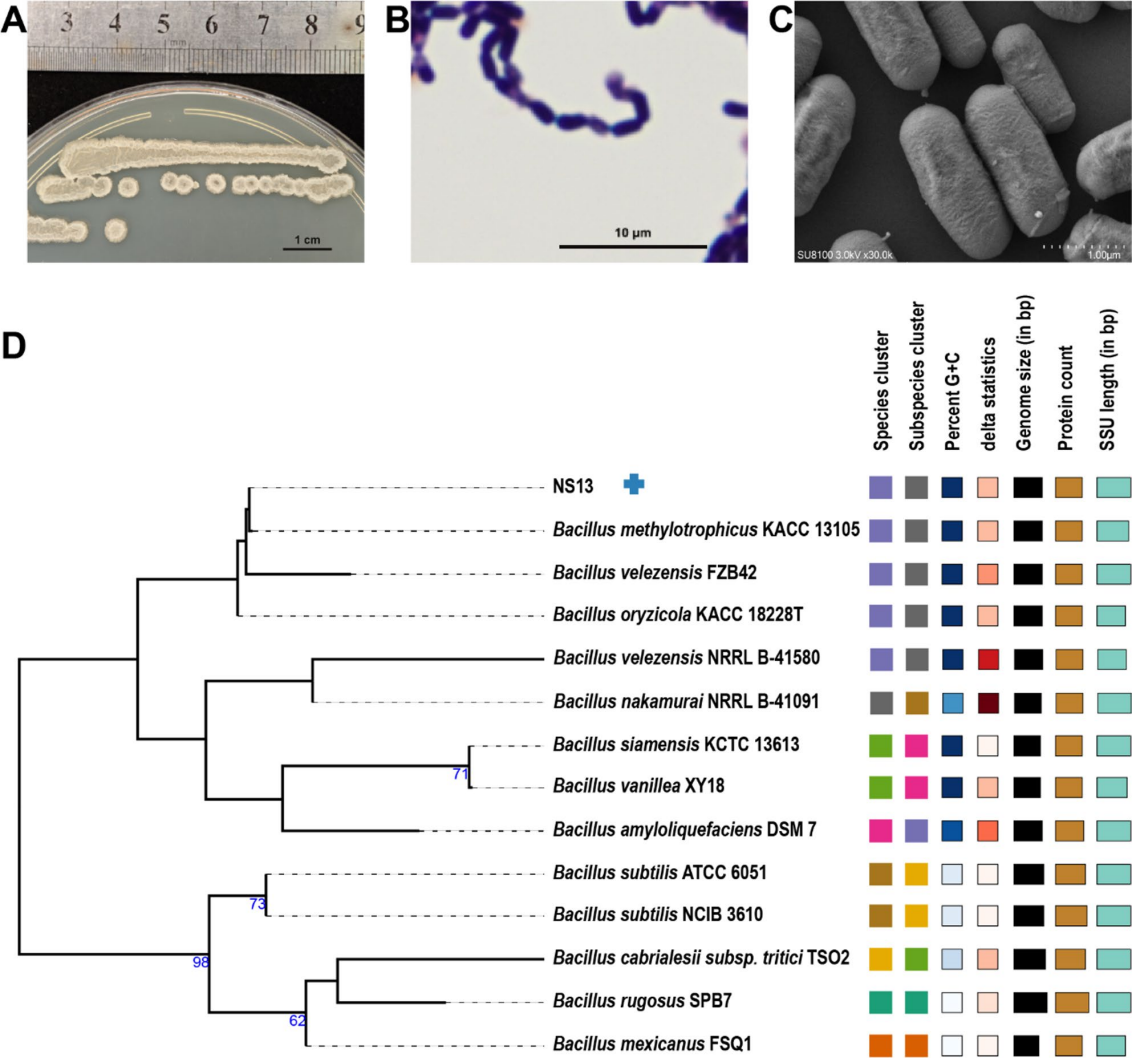
Morphological characteristics and phylogenetic tree of strain NS13 based on 16S rRNA gene sequences. **A** Colony morphology. **B** Gram staining. **C** SEM image. **D** Phylogenetic tree based on 16S rRNA

Endophytic bacteria were isolated from *L. macranthoides* root tissues, and a strain with distinct morphological characteristics, resembling *Bacillus*, was selected after three rounds of purification and designated NS13 (Fig. 2A). Colonies on LB agar were round, opaque, creamy white, with smooth surfaces and irregular margins. Microscopically, cells were rod-shaped (approximately 0.8–1.0 × 2.5–3.5 μm), motile, and capable of forming endospores. Gram staining confirmed that NS13 was Gram-positive (Fig. 2B, C), consistent with the typical morphology of *Bacillus* species. A phylogenetic tree based on 16S rRNA gene sequences (Fig. 2D) showed a close relationship with the type strain *B. velezensis* FZB42. This taxonomic classification was supported by physiological and biochemical characteristics (Table S1): NS13 was negative for the Voges-Proskauer test, positive for nitrate reduction, capable of utilizing citrate and propionate as carbon sources, but unable to metabolize D-mannitol, D-xylose, or L-arabinose. It exhibited protease activity (gelatin liquefaction), starch hydrolysis, weak salt tolerance (up to 7% NaCl), and growth at pH 5.7.

### Plate confrontation assay of NS13 against *Fusarium* species

**Fig. 3:**
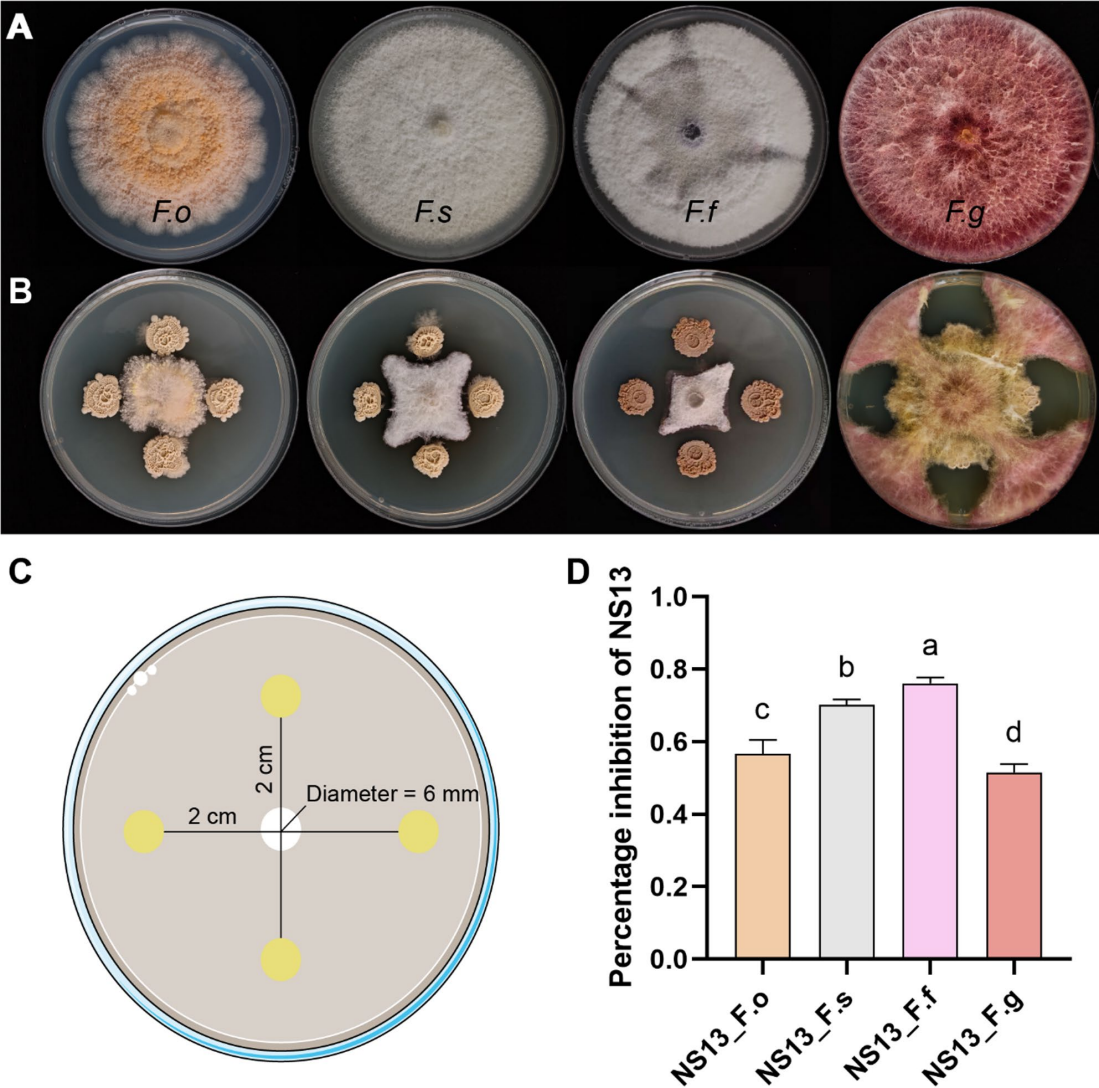
Antagonistic activity of NS13 against four *Fusarium* strains. **A** Individual cultures of four *Fusarium* strains. **B** Plate confrontation of NS13 with four *Fusarium* strains. **C** Schematic of the confrontation assay. **D** Percentage inhibition of NS13 against four *Fusarium* strains. Bars represent means ± standard error of the means(mean ± standard error of the mean, SEM). Different letters indicate statistically significant differences between treatment groups (*p* < 0.05)

Plate confrontation assay (Fig. 3C) demonstrated significant inhibition of *F. oxysporum* (from *L. macranthoides* root rot) by NS13, with an inhibition rate of 56.60% ± 3.88%. In pairwise confrontation assays with three other *Fusarium* strains, NS13 formed clear inhibition zones, with morphological changes observed at the fungal colony edges, suggesting the production of potent antifungal metabolites (Fig. 3A, B). Quantitative analysis (Fig. 3D) showed varying inhibition rates: 76.04% ± 1.68% against *F. fujikuroi* (highest), 70.14% ± 1.46% against *F. solani*, 56.60% ± 3.88% against *F. oxysporum*, and 51.59% ± 2.24% against *F. graminearum*.

### Genomic features and taxonomic analysis of NS13

**Fig. 4:**
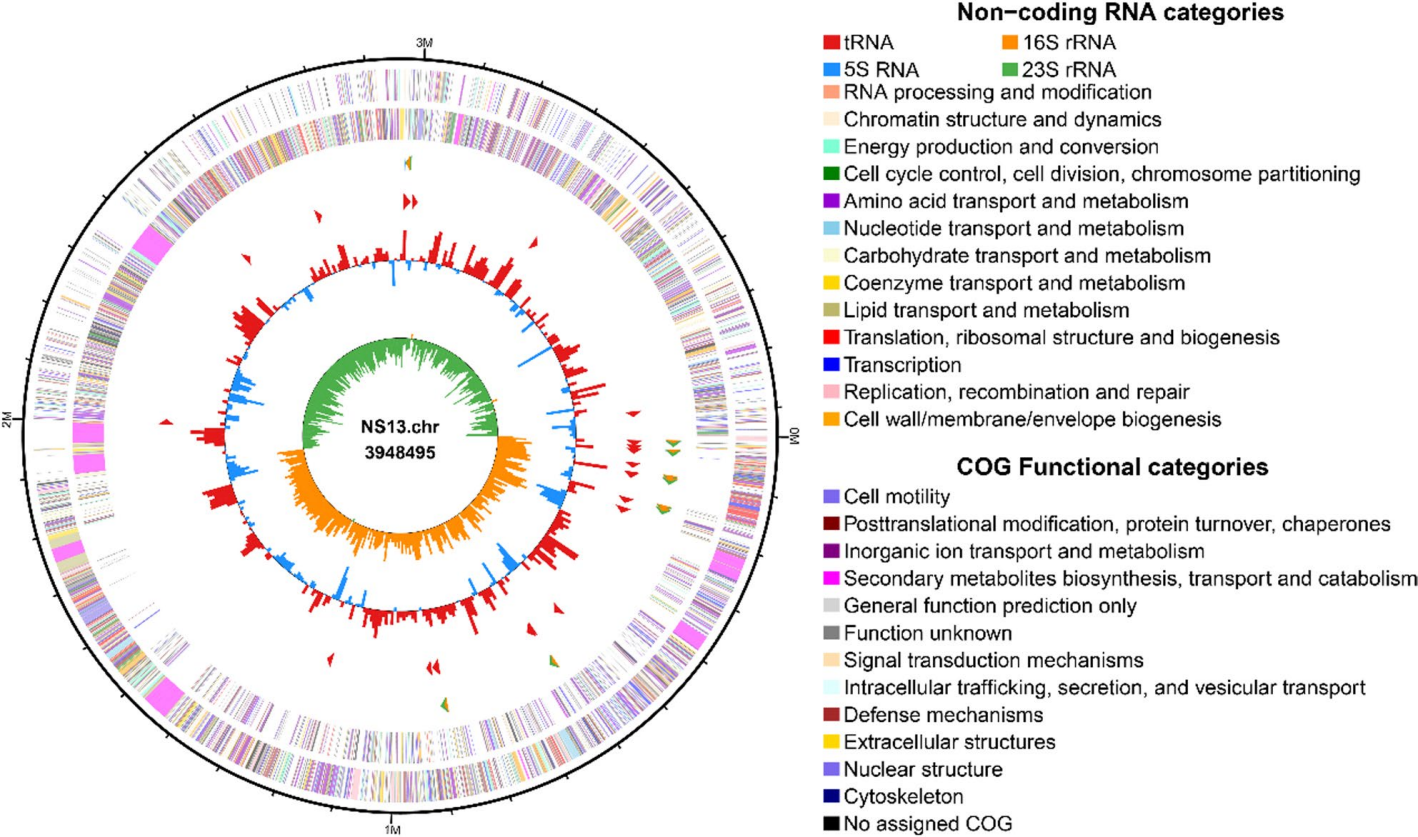
Genomic circular map of NS13. Note: From outer to inner rings, the outermost ring indicates genome size; the second and third rings represent CDS on the positive and negative strands, with colors indicating different COG functional categories; the fourth ring shows rRNA and tRNA; the fifth ring displays GC content, with outward red peaks indicating regions above the average GC content and inward blue peaks indicating regions below; the innermost ring shows GC skew (G-C/G + C), with positive values favoring positive-strand transcription and negative values favoring negative-strand transcription

Hybrid assembly using PacBio RSIII and Illumina NovaSeq 6000 data yielded a complete, closed circular chromosome for *B. velezensis* NS13 (Fig. 4), with a genome size of 3.95 Mb and a GC content of 46.55%. A total of 4,060 protein-coding genes were predicted (Table S2), with 91.26% annotated in the NR database (Table S3), indicating robust metabolic capacity and environmental adaptability. Functional annotation revealed potential antifungal mechanisms: the CAZy database (Figure S2A) identified 152 carbohydrate-active enzyme genes, including GH1, GT2, and CBM50 families, suggesting capabilities for degrading complex carbon sources and recognizing fungal cell wall components, enhancing direct fungal inhibition. KEGG annotation identified 3,463 metabolism-related genes (Figure S2B-C), with significant enrichment in secondary metabolite biosynthesis (24.09%) and antibiotic biosynthesis (18.16%), indicating the ability to produce diverse antifungal molecules. Genes related to amino acid synthesis (10.72%) and ABC transport systems (10.1%) supported metabolite synthesis, regulation, and transport. COG annotation (Figure S3A) showed enrichment in amino acid transport/metabolism, carbohydrate metabolism, and cell wall biogenesis, aiding activity under fungal stress. GO annotation (Figure S3B) highlighted widespread “metabolic process” and “catalytic activity” genes, reflecting diverse metabolic and environmental response potential. Collectively, NS13’s genetic repertoire supports carbon utilization, secondary metabolite production, transmembrane transport, and fungal recognition, forming the basis of its antifungal activity.

Phylogenomic analysis confirmed NS13’s taxonomic status. A 16S rRNA-based phylogenetic tree (Fig. 2D) showed a close relationship with *B. velezensis*. A whole-genome-based phylogenetic tree (Figure S3A) with multiple reference strains confirmed NS13’s closest relation to FZB42. isDDH and ANI analyses (Figure S3B) showed similarity values exceeding 70% and 95%, respectively, meeting species delineation criteria. Based on morphological, physiological, biochemical, and phylogenomic evidence, NS13 was classified as *Bacillus velezensis* NS13 within the Firmicutes, Bacilli, Bacillales, Bacillaceae, and Bacillus genera.

### Mining of antifungal genes in NS13

**Fig. 5:**
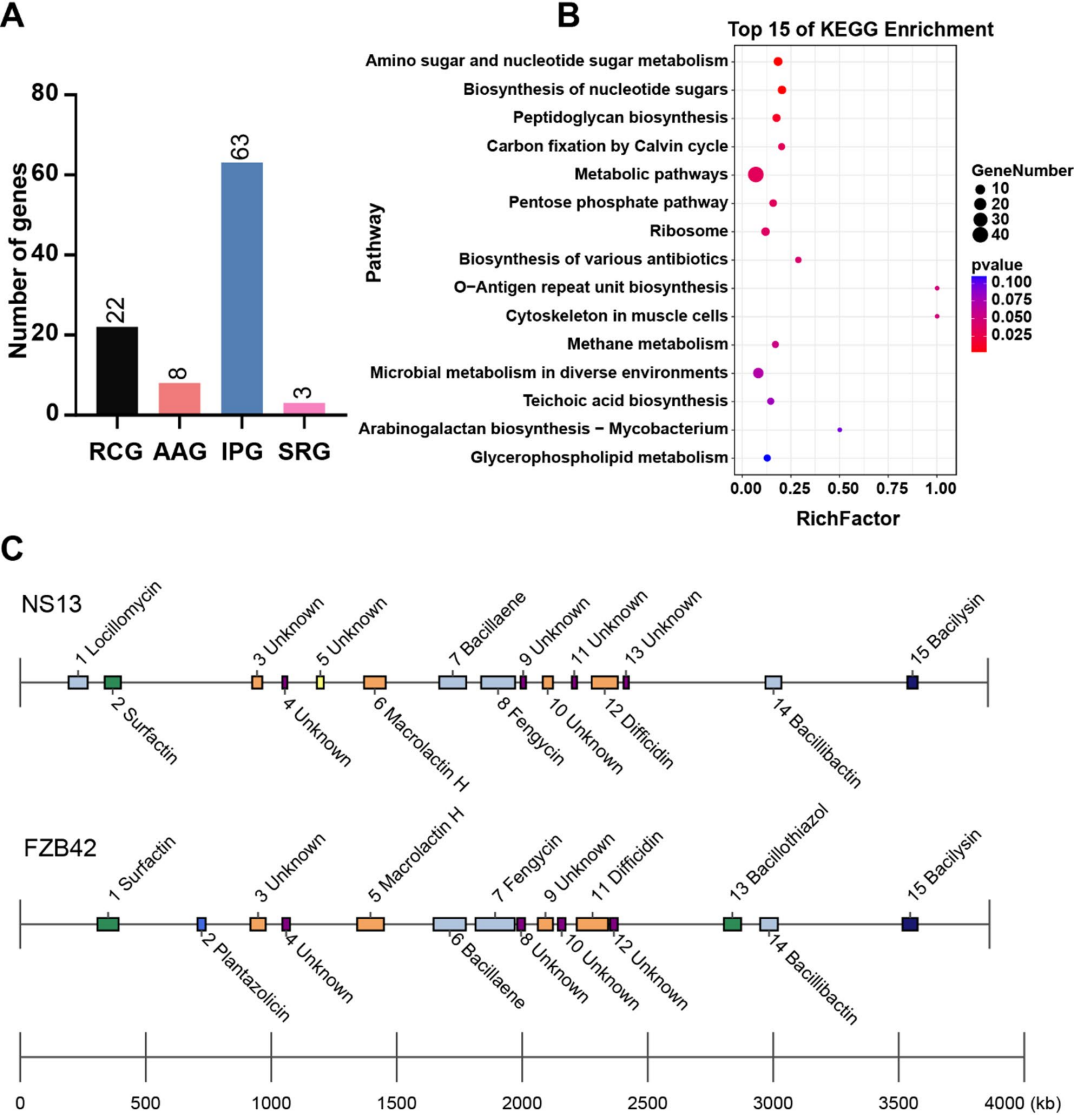
Biocontrol characteristics of NS13 genome. **A** Statistics of four biocontrol gene categories. **B** Heatmap of gene-biocontrol mechanism associations. **C** Secondary metabolite BGC analysis

Functional annotation revealed a systematic antifungal genetic foundation in *B. velezensis* NS13, with 96 biocontrol-related genes identified (Fig. 5A): 65.63% induced resistance genes, 22.92% resource competition genes, 8.33% antibacterial activity genes, and 3.13% stress-resistance genes. KEGG enrichment analysis (Fig. 5B) identified 15 significant pathways, including antibiotic synthesis, two-component systems, and ABC transport, supporting NS13’s antifungal mechanisms via direct antibiosis, nutrient competition, and induced resistance.

AntiSMASH analysis identified 15 secondary metabolite BGCs (Fig. 5C), spanning 888.062 kb (22.49% of the genome). Eight BGCs had defined functions (Table S6), encoding antifungal metabolites like fengycin, surfactin (lipopeptides), bacillaene, difficidin (polyketides), bacillibactin (siderophore), and bacilysin (membrane-disrupting). Fengycin and surfactin disrupt fungal membranes, bacillaene inhibits chitin synthesis, and bacillibactin limits fungal iron acquisition [28, 40]. Seven unannotated BGCs (22.04%) contained PKS-NRPS hybrid modules and unique modification enzyme domains, suggesting potential for novel antifungal compounds. Comparison with FZB42 revealed a unique locillomycin BGC in NS13, indicating broader or differential antifungal capabilities.

In summary, NS13’s biocontrol genes, diverse BGCs, and detectable broad-spectrum antifungal metabolites synergistically contribute to direct antibiosis, nutrient competition, and induced resistance, forming the genetic and metabolic basis for its effective antagonism against *Fusarium* species.

### LC-MS analysis reveals antifungal metabolites in NS13

High-resolution mass spectrometry confirmed NS13’s rich antifungal metabolite profile. Multiple compounds with potential biocontrol activity were detected (Table S7), including cyclic dipeptides with a diketopiperazine (DKP) scaffold, such as Cyclo(phenylalanyl-prolyl), 3-(1-hydroxyethyl)−2,3,6,7,8,8a-hexahydropyrrolo[1,2-a]pyrazine-1,4-dione, 3-(propan-2-yl)-octahydropyrrolo[1,2-a]pyrazine-1,4-dione, and 3-[(4-hydroxyphenyl)methyl]-octahydropyrrolo[1,2-a]pyrazine-1,4-dione. Additionally, fatty acid amides with membrane-disrupting potential, such as oleamide, erucamide, and stearamide, were detected [41–43]. Active dipeptides like carnosine and valylproline, capable of scavenging free radicals and modulating microbial interactions, were also identified, along with phenolic antioxidants like rutin and quercetin-3β-D-glucoside, reported to inhibit fungal virulence factors [44, 45], potentially enhancing NS13’s resilience to fungal stress. Further confirmation of their biological functions through structural analysis and activity validation is needed.

## Discussion

### Genomic analysis reveals the genetic basis of NS13 biocontrol

Genomic analysis serves as a powerful methodological tool, providing rich insights into the biocontrol mechanisms of *Bacillus velezensis* [46–48]. These mechanisms, including the synthesis of antibacterial compounds, competitive nutrient suppression, and induction of host resistance, are all reflected at the genomic level [49–51]. Furthermore, genomic analysis offers critical support for the genetic improvement of *Bacillus velezensis*, enabling optimization of its antifungal activity or environmental adaptability through gene editing or synthetic biology approaches, thereby enhancing its biocontrol application potential [52, 53].

Specifically, the 3.95 Mb NS13 genome encodes 4,060 proteins, including 96 antifungal-related genes. Specifically (Table S5), polyketide synthase genes (e.g., K15327) synthesize antifungal polyketides [40], endoglucanase genes (K01179) degrade fungal cell walls [54], and siderophore transport genes (K23188) limit fungal iron acquisition [55]. Two-component system histidine kinases (K07777) and Fur iron regulators (K03711) may induce host resistance via JA/SA signaling and oxidative stress responses [56], while sporulation genes (K06378) support rhizosphere colonization and sustained biocontrol [44]. Notably, NRPS and PKS genes, such as those responsible for fengycin and bacillaene synthesis, align with known antifungal mechanisms in *B. velezensis* [28, 57]. Siderophore genes, including those for bacillibactin, support nutrient competition by limiting *Fusarium* iron acquisition [46]. Genes linked to induced systemic resistance, such as those regulating JA/SA signaling, suggest enhanced host defense, consistent with other plant [47]. The discovery of seven uncharacterized BGCs encoding novel PKS-NRPS hybrid enzymes highlights NS13’s potential to produce unique antifungal compounds, distinguishing it from FZB42. This genetic diversity positions NS13 as a valuable resource for biocontrol and novel metabolite discovery.

### Metabolomics provides the material basis for NS13’s antifungal activity

Metabolomics, as a core technical tool for analyzing microbial functional metabolites, can systematically identify various antifungal active components produced by *Bacillus velezensis* during its growth and metabolism, thereby providing direct evidence for clarifying the material basis of its biocontrol effects [48]. LC-MS/MS analysis identified a range of NS13 antifungal metabolites, including cyclic dipeptides [49], fatty acid amides such as erucamide, and triterpenoids such as oleanolic acid. These cyclic dipeptides, known for membrane permeability and structural stability, are widely recognized for antibacterial and antifungal activity [50, 51], and their repeated detection suggests they are key factors in NS13’s inhibition of *Fusarium*. Cyclic dipeptides disrupt fungal signaling and membrane integrity, while lipopeptides like fengycin and surfactin destabilize fungal membranes [58]. The detection of oleanolic acid [59], which is known for its antifungal properties, suggests synergy with lipopeptides, enhancing NS13’s biocontrol efficacy. Notably, polyamines like spermine were detected, which stabilize DNA, regulate membrane permeability [53], and activate plant immune signaling, suggesting both direct antifungal activity and enhanced host defense. In addition, triterpenoids with potential antifungal activity, such as oleanolic acid [57], and some metabolites that have not been fully annotated, such as PPK and NP-011220, were also detected, which may represent secondary metabolites unique to NS13. Uncharacterized BGCs encoding novel metabolites further underscore NS13’s potential for developing new antifungal agents. Compared to other *Bacillus* strains, NS13’s unique production of locillomycin and diverse dipeptides suggests broader antifungal capabilities, potentially overcoming strain-specific efficacy limitations.

These findings align with prior studies demonstrating *B. velezensis* efficacy against *Fusarium* diseases in crops like wheat and maize [60, 61]. For instance, FZB42 produces similar lipopeptides, including fengycin and surfactin, to suppress *F. graminearum* [62]. However, NS13’s unique locillomycin [63] BGC and higher BGC diversity suggest enhanced antifungal potential, particularly against *F. fujikuroi* and *F. solani.* Unlike earlier studies focusing on cereals, this work extends *B. velezensis* application to medicinal plants, addressing a critical gap in *L. macranthoides* root rot control.

Differences in inhibition rates, such as lower efficacy against *F. graminearum* compared to *F. fujikuroi*, may reflect species-specific susceptibility or experimental conditions, including medium and incubation time. The integrated genomic and metabolomic approach distinguishes this study from earlier phenotype-based work, providing mechanistic insights into NS13’s biocontrol efficacy.

### Potential and challenges of *B. velezensis* NS13 in controlling *L. macranthoides* root rot

*Fusarium* species represent a major group of soil-borne pathogens that cause devastating economic losses worldwide by inducing wilt, rot, and blight diseases across diverse crops [5, 12]. In medicinal plants, *Fusarium*-induced root rot severely reduces biomass and bioactive compound yields, threatening both agricultural productivity and the quality of herbal products [16–18]. For instance, *F. oxysporum* and *F. solani* are responsible for significant yield losses in *Lonicera* species and other economically important crops such as tomato, cucumber, and banana [6, 9, 11].

The genus *Bacillus*, particularly *B. velezensis*, has emerged as a promising biocontrol agent due to its ability to produce diverse antimicrobial compounds, promote plant growth, and induce systemic resistance in hosts [26, 31, 32]. Previous studies have reported *B. velezensis* FZB42 and related strains effectively suppressing *Fusarium graminearum* in wheat and *F. oxysporum* in tomato through the production of fengycin, surfactin, and difficidin [28, 30]. Consistent with these findings, NS13 exhibited strong antagonistic activity against multiple *Fusarium* species and encoded similar biosynthetic gene clusters (fengycin, surfactin, bacillaene, bacillibactin, and bacilysin). Notably, the presence of a unique locillomycin cluster and seven uncharacterized BGCs in NS13 expands the known metabolic repertoire of *B. velezensis*, suggesting potential for discovering new antifungal molecules [53, 63]. These results align with prior reports that *Bacillus*-based biocontrol agents offer an eco-friendly and cost-effective alternative to chemical fungicides [20, 43, 57]. Therefore, NS13 not only contributes mechanistic insights into *Bacillus–Fusarium* interactions but also provides an economically and environmentally sustainable solution for managing root rot in *L. macranthoides* and potentially other crops.

In root rot-affected *L. macranthoides* rhizospheres, *Fusarium* enrichment aligns with its established role as a major soil-borne pathogen. The observed decline in beneficial fungi such as *Trichoderma* and in microbial diversity, as indicated by Chao1 and Shannon indices with *p* < 0.05, points to microbial dysbiosis, likely driven by *F. oxysporum* mycotoxins such as fumonisins that suppress competing microbes [64]. The high *Fusarium* abundance in diseased plants confirms its role as the primary driver of root rot, consistent with its pathogenicity in related species like *L. japonica*. These findings highlight the need for targeted biocontrol to restore microbial balance and suppress *Fusarium* dominance.

B. velezensis NS13 exhibited potent antagonism against *F. oxysporum* with 56.60% inhibition and against other *Fusarium* species, with the highest efficacy against *F. fujikuroi* at 76.04%. This variability may reflect differences in cell wall composition or metabolic vulnerabilities among *Fusarium* species [5]. Clear inhibition zones in confrontation assays suggest diffusible antifungal compounds, supported by the detection of lipopeptides such as fengycin and surfactin, as well as polyketides such as bacillaene, which are known to disrupt fungal membranes and spore germination [62]. Unlike chemical fungicides, which face resistance issues, NS13’s multi-mode action minimizes resistance development. Differential inhibition rates suggest NS13’s efficacy may be optimized for specific Fusarium strains, warranting field validation.

This study elucidates the genetic and metabolic mechanisms of NS13’s antagonism against *Fusarium* species, positioning it as a viable alternative to chemical fungicides and addressing environmental concerns like pesticide residues and resistance [62]. Practically, NS13 could be developed as a biofungicide for sustainable *L. macranthoides* cultivation, reducing yield losses and ensuring medicinal quality. The discovery of novel BGCs opens avenues for identifying new antifungal compounds with potential in medicine and agriculture.

Despite its strengths, this study has limitations. Plate-based assays, while reliable, may not fully reflect NS13’s performance in complex soil environments, where abiotic factors such as pH and humidity, along with microbial interactions, may modulate efficacy. Field trials are needed to validate NS13’s biocontrol potential under natural conditions. The functions of seven uncharacterized BGCs remain speculative, requiring targeted gene knockout or heterologous expression studies for confirmation. The focus on *L. macranthoides* limits direct extrapolation to other crops, though NS13’s broad-spectrum activity suggests wider applicability. Future research should explore NS13’s interactions with plant immune systems and its long-term stability in rhizosphere microbiomes. Overall, this study establishes *B. velezensis* NS13 as a potent biocontrol agent against *Fusarium*-induced root rot in *L. macranthoides*, leveraging synergistic biocontrol genes and antifungal metabolites. Its application could transform disease management in medicinal plant cultivation, promoting sustainability. Future studies should focus on field validation, functional characterization of novel BGCs, and NS13’s role in modulating plant immunity to enhance biocontrol efficacy.

## Conclusions

This study provides comprehensive insights into the microbial dynamics associated with *Fusarium*-induced root rot in *Lonicera macranthoides* and the biocontrol potential of *Bacillus velezensis* strain NS13. We observed significant rhizosphere microbial dysbiosis in diseased plants, characterized by an increase in pathogenic fungi such as *Fusarium* and *Plectosphaerella*, along with a decrease in beneficial fungi including *Trichoderma*, highlighting the ecological imbalance contributing to disease progression. The endophytic strain NS13, isolated from healthy roots, exhibited strong antagonistic activity against multiple *Fusarium* species, including *F. oxysporum*, *F. solani*, *F. graminearum*, and *F. fujikuroi*. Genomic analysis revealed 96 biocontrol-related genes and 15 secondary metabolite biosynthetic gene clusters, of which five are associated with antifungal activity, three with antibacterial activity, and seven potentially encode novel bioactive compounds. LC–MS/MS metabolomics confirmed the presence of antifungal metabolites, including cyclic dipeptides, fatty acid amides (e.g., erucamide), and oleanolic acid, corroborating the strain’s antagonistic mechanisms at the molecular level. Together, these findings illuminate the complex soil–plant–microbe interactions underlying root rot and reveal the multifaceted biocontrol mechanisms of NS13.

Looking forward, *B. velezensis* NS13 holds significant promise as a sustainable biocontrol agent. Its rich repertoire of bioactive metabolites not only provides opportunities for the discovery of novel antifungal compounds but also lays the foundation for environmentally friendly strategies to manage *Fusarium* pathogens in medicinal plants. Future research should focus on large-scale field trials, formulation development, and optimization of application strategies to fully harness the potential of NS13 in sustainable agriculture. Moreover, the identification of novel secondary metabolite clusters opens avenues for bioengineering and natural product discovery, expanding the utility of NS13 beyond biocontrol into pharmaceutical and agro-biotechnological applications.

## Supplementary Information

Supplementary Material 1.

Supplementary Material 2.

Supplementary Material 3.

## Data Availability

The datasets generated and/or analysed during the current study are available in the National Center for Biotechnology Information (NCBI) repository, primary accession code PRJNA1306347.

## Abbreviations

ITS: Internal Transcribed Spacer
LB: Luria-Bertani
OD: Optical Density
PCR: Polymerase Chain Reaction
SEM: Scanning Electron Microscopy
