# Supplementary material for: Integrated genomic and metabolomic analysis reveals the biocontrol potential of endophytic Bacillus velezensis NS13 against Fusarium species in Lonicera macranthoides
Source: BMC Microbiol. 2026 Jan 5;26:5. doi: 10.1186/s12866-025-04551-x (PMC12771994; doi:10.1186/s12866-025-04551-x)
Supplement: Supplementary file 3 — Supplementary Material 3. [file 12866_2025_4551_MOESM3_ESM.docx]

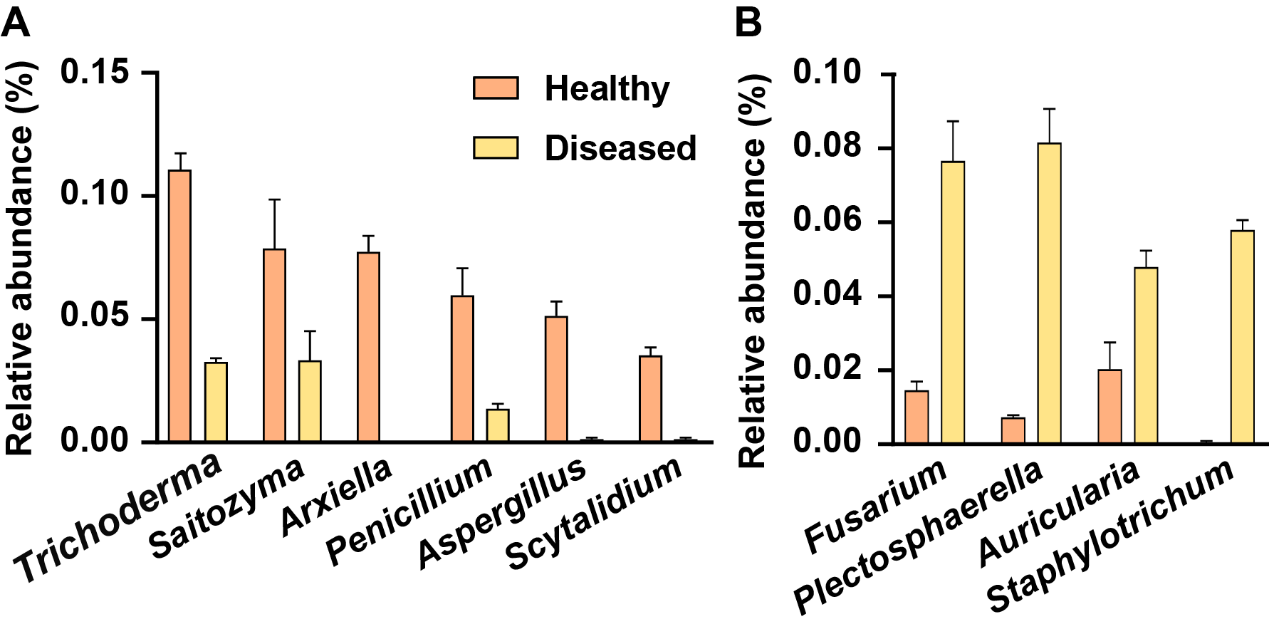


Fig. S1 Quantitative analysis of the composition of rhizosphere microbial genera in Lonicera japonica plants at the level of health and root rot disease(A) Downregulation in health(B) Upregulation in diseases


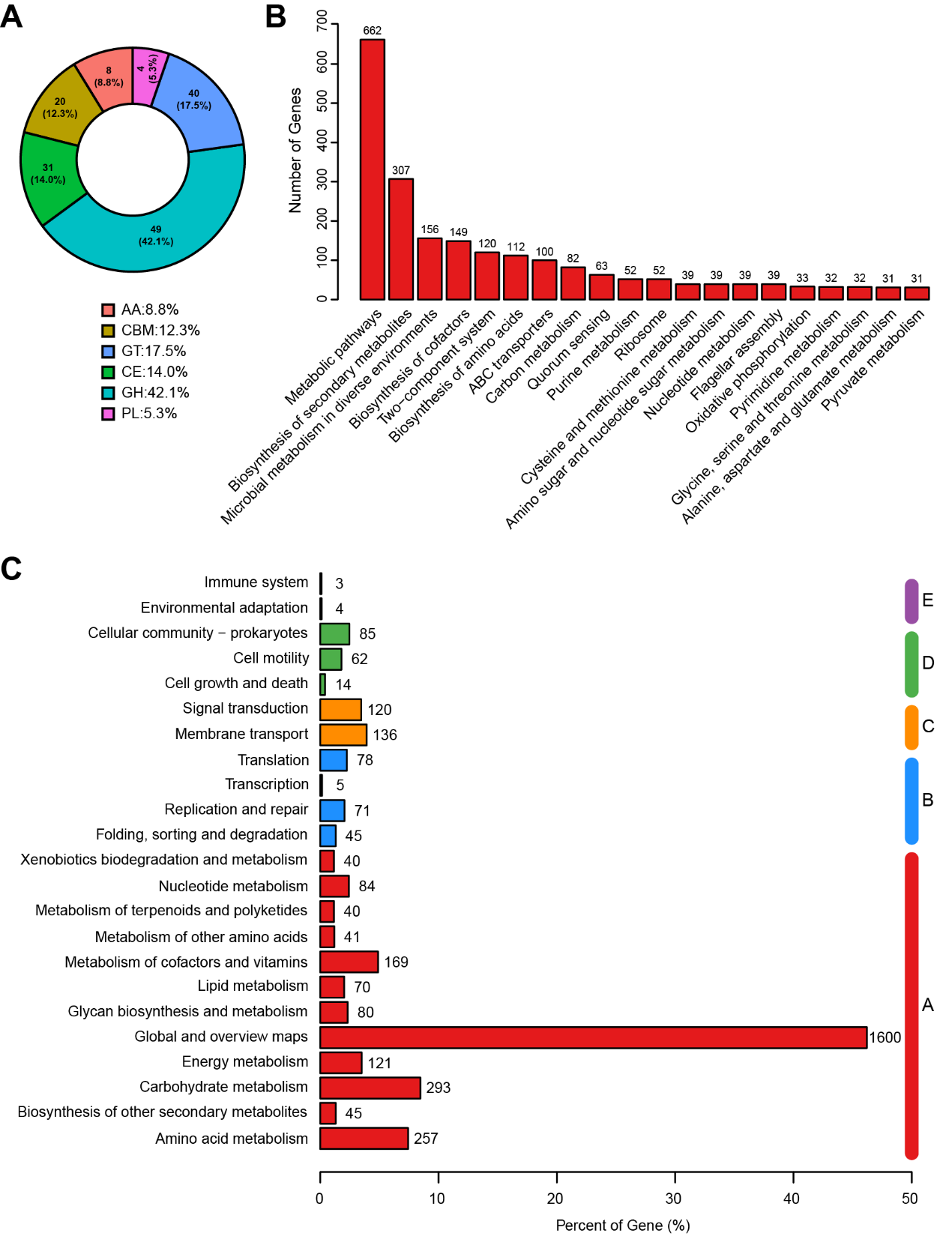


Fig. S2 Annotations of CAZyme and KEGG databases for the NS13 genome (A) The number of CAZyme types. (B-C) KEGG database annotation.


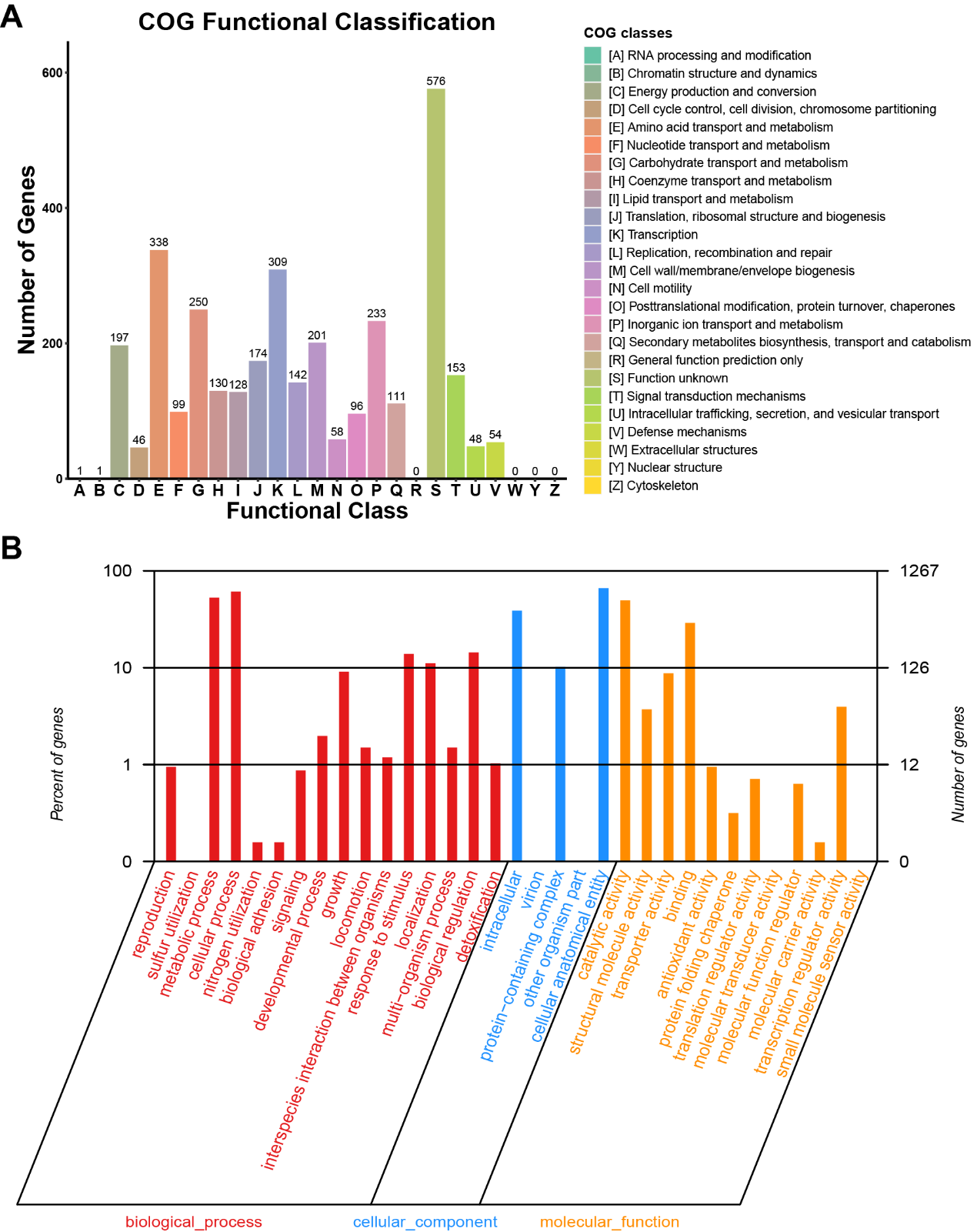


Fig. S3 Annotations of COG and GO databases for the NS13 genome (A) COG annotation. (B) GO database annotation.


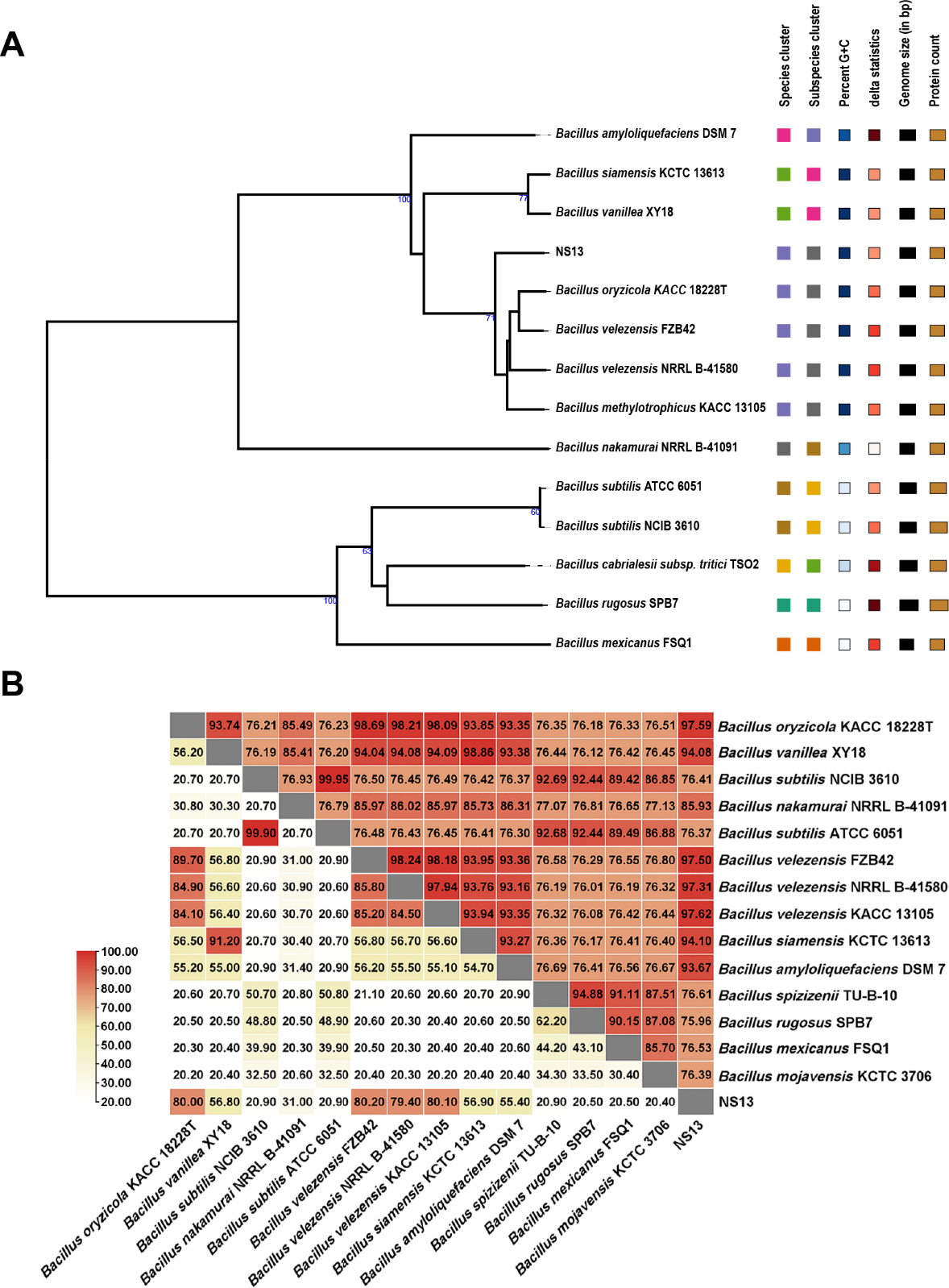


Fig. S4 Phylogenetic analysis of NS13. (A) NS13 genome level evolutionary tree (B): isDDH analysis and ANI analysis of NS13.

Table S1 The fungal reference strains and bacterial agents used in this study

| Genus name | Host | Disease type | Strain number |
| --- | --- | --- | --- |
| Bacillus velezensis | *Lonicera macranthoides* | - | NS13 |
| Fusarium oxysporum | *Lonicera macranthoides* | Root rot | Y1 |
| Fusarium fujikuroi | *Avena fatua* L. | Spike rot | BNCC186247 |
| Fusarium solani | *Dendrobium* Sw. | Root rot | BNCC121547 |
| Fusarium graminearum | *Triticum aestivum* L. | FHB | BNCC113713 |

Table S2 Physiological and biochemical characteristics of strain NS13

| Biochemical testing | NS13 |
| --- | --- |
| V-P | - |
| Citrate utilization | + |
| Propionate Utilization | + |
| D-Xylose Utilization | - |
| L-arabinose | - |
| Mannitol Fermentation | - |
| Gelatin Liquefaction | + |
| 7% NaCl tolerance | - |
| PH 5.7 growth capacity | + |
| Nitrate Reduction | + |
| Starch Hydrolysis | + |

Table S3 Genomic component analysis of strain NS13

|  | NS13 |
| --- | --- |
| Gene num | 4060 |
| Gene total length | 3559779 |
| Gene average length | 876 |
| Gene density genes per kb | 1.028 |
| GC content in gene region | 47.2% |
| Gene/Geonme | 90.2% |
| plus Gene num | 1932 |
| minus Gene num | 2128 |

Table S4 Genomic annotation of strain NS13

|  | NS13 |
| --- | --- |
| Total protein | 4060 |
| NR | 3705 |
| GO | 1267 |
| COG | 3044 |
| KEGG | 2328 |
| Swiss | 3413 |
| CAZy | 152 |
| CARD | 120 |

Table S6 Secondary metabolite gene cluster of NS13

| Cluster number | Compound | Type | From | To | Size(kb) |
| --- | --- | --- | --- | --- | --- |
| 1 | Locillomycin | NRPS, transAT-PKS | 197735 | 275463 | 77.729 |
| 2 | Surfactin | NRPS | 345048 | 410455 | 65.408 |
| 3 | Unknown | PKS-like | 946326 | 987570 | 41.245 |
| 4 | Unknown | terpene | 1069592 | 1090332 | 20.741 |
| 5 | Unknown | lanthipeptide-class-ii | 1210279 | 1239168 | 28.89 |
| 6 | Macrolactin H | transAT-PKS | 1403124 | 1491417 | 88.294 |
| 7 | Bacillaene | transAT-PKS,T3PKS,NRPS-like,NRPS | 1710102 | 1820234 | 110.133 |
| 8 | Fengycin | NRPS,betalactone | 1881285 | 2019115 | 137.831 |
| 9 | Unknown | terpene | 2041756 | 2063639 | 21.884 |
| 10 | Unknown | T3PKS | 2132288 | 2173394 | 41.107 |
| 11 | Unknown | terpene-precursor | 2250746 | 2271708 | 20.963 |
| 12 | Difficidin | transAT-PKS | 2332437 | 2438603 | 106.167 |
| 13 | Unknown | terpene-precursor | 2461898 | 2482788 | 20.891 |
| 14 | Bacillibactin | terpene-precursor,NRP-metallophore,NRPS,RiPP-like | 3040880 | 3106239 | 65.36 |
| 15 | Bacilysin | other | 3620234 | 3661652 | 41.419 |
